# Whole Genome Expression Profiling Shows that BRG1 Transcriptionally Regulates UV Inducible Genes and Other Novel Targets in Human Cells

**DOI:** 10.1371/journal.pone.0105764

**Published:** 2014-08-26

**Authors:** Ling Zhang, Leah Nemzow, Hua Chen, Jennifer J. Hu, Feng Gong

**Affiliations:** Department of Biochemistry and Molecular Biology, University of Miami Miller School of Medicine, Miami, Florida, United States of America; Florida International University, United States of America

## Abstract

UV irradiation is known to cause cyclobutane pyrimidine dimers (CPDs) and pyrimidine (6–4) pyrimidone photoproducts (6-4PPs), and plays a large role in the development of cancer. Tumor suppression, through DNA repair and proper cell cycle regulation, is an integral factor in maintaining healthy cells and preventing development of cancer. Transcriptional regulation of the genes involved in the various tumor suppression pathways is essential for them to be expressed when needed and to function properly. BRG1, an ATPase catalytic subunit of the SWI/SNF chromatin remodeling complex, has been identified as a tumor suppressor protein, as it has been shown to play a role in Nucleotide Excision Repair (NER) of CPDs, suppress apoptosis, and restore checkpoint deficiency, in response to UV exposure. Although BRG1 has been shown to regulate transcription of some genes that are instrumental in proper DNA damage repair and cell cycle maintenance in response to UV, its role in transcriptional regulation of the whole genome in response to UV has not yet been elucidated. With whole genome expression profiling in SW13 cells, we show that upon UV induction, BRG1 regulates transcriptional expression of many genes involved in cell stress response. Additionally, our results also highlight BRG1's general role as a master regulator of the genome, as it transcriptionally regulates approximately 4.8% of the human genome, including expression of genes involved in many pathways. RT-PCR and ChIP were used to validate our genome expression analysis. Importantly, our study identifies several novel transcriptional targets of BRG1, such as *ATF3*. Thus, BRG1 has a larger impact on human genome expression than previously thought, and our studies will provide inroads for future analysis of BRG1's role in gene regulation.

## Introduction

SWI/SNF is part of a family of chromatin remodeling complexes which act as master regulators of gene expression in yeast and human cells [1], by modifying nucleosomes in an ATP-dependent fashion. Mammalian SWI/SNF complexes contain one of two ATP catalytic subunits, BRM (Brahma) or BRG1 (Brahma Related Gene), and also contain additional proteins called BAFs (BRM/BRG1 Associated Factors) consisting of core and accessory subunits [2], which function as a diverse array of biochemical and functional activities [3]–[6].

Several lines of evidence suggest that the SWI/SNF chromatin remodeling complexes are important for protection of genome integrity. Previous studies have shown SWI/SNF accumulation at DNA double strand breaks in yeast [7], [8]. In addition, SWI/SNF is involved in chromatin dynamics after exposure to ultraviolet (UV) radiation [9], [10] and appears to be associated with Rad4-Rad23, a heterodimer that recognizes UV lesions in yeast and facilitates nucleotide excision repair (NER) for such lesions [11]. Interestingly, it has been shown that the BAF subunits in SWI/SNF-like complexes play a protective role in genome integrity, since inactivation of the SWI/SNF-like BAF complexes renders human cells sensitive to DNA damaging agents, such as UV and ionizing radiation (IR) [12], [13]. BRG1 in particular, appears to be a crucial part of SWI/SNF-like BAF complexes, since expression of a dominant negative mutant of BRG1 inactivates of the BAF complexes and results in inefficient DNA double-strand break repair and sensitizes cells to ionizing radiation [14]. Cells lacking functional BRG1 are sensitive to various DNA damage agents, including ionizing radiation and doxorubicin [14], cisplatin [15], [16], and UV radiation [12]. BRG1 has also been shown to play a role in genome integrity by contributing to proper NER of CPDs induced by UV irradiation [17], [18], and has been shown to also respond to DNA damage by suppressing UV induced apoptosis, and restoring checkpoint deficiency [12].

Defects in DNA repair and improper regulation of cell cycle progression and apoptosis can lead to development of cancer, and thus much recent work has focused on the role of the BAF ATPase BRG1 in cancer [19]–[21]. *BRG1* gene is frequently deleted or mutated in a variety of tumor cell lines, implicating *BRG1* as a potential tumor suppressor gene [22], [23], and mouse models have confirmed the tumor suppressor activities of BRG1 [20], [24], [25]. Given that transcriptional response is a well-documented strategy for cells to survive exposure to various DNA damaging agents [26]–[28], thereby suppressing tumor formation, it becomes important to understand how BRG1 may play a role in regulating genes responsible for DNA repair and cell cycle progression while improving our efforts in combating cancer.

Previous whole genome analyses have shown that BRG1 transcriptionally regulates genes involved in cellular proliferation and tumor suppression [29], [30]. Yet, only a limited number of genes regulated by BRG1 in response to UV radiation have been reported, and the impact of UV induced gene regulation by BRG1 on the human transcriptome as a whole is not well understood. In this study, we investigated the role of BRG1 in the transcriptional response to UV radiation with whole genome expression studies. Here we use a microarray approach to systematically compare UV-induced gene expression profiles in two isogenic cell lines derived from the adrenal cortical carcinoma cell line SW13, which lack BRG1 protein expression. Experiments with triplicates were designed to assess BRG1-mediated alterations in over 47,000 transcripts in response to UV radiation. Reverse transcriptase polymerase chain reaction (RT-PCR) was performed to confirm microarray data, and Chromatin Immuniprecipitation (ChIP) showed that BRG1 associated with promoter regions of these regulated genes. Here we show that BRG1 does indeed regulate transcription of genes important for UV damage repair and cell signaling upon UV induction, and identify several novel BRG1 transcriptional targets. These results will direct future experiments to fully understand BRG1's role as a tumor suppressor.

## Materials and Methods

### Cell lines and culture

Stable cell lines, SW13+pREP7 (vector), SW13+pREP7+BRG1 from previous study in which BRG1 isoform C expression has been confirmed [12], and SW13 and 293T cell lines (purchased from the ATCC), were cultured in Dulbecco's modified Eagle's medium (DMEM) (CELLGRO, Manassas, VA) supplemented with 10% fetal bovine serum (HyClone, Logan, UT) and penicillin-streptomycin.

### UV Treatment and 5-aza-2′-deoxycytidine treatment

**Figure 1:**
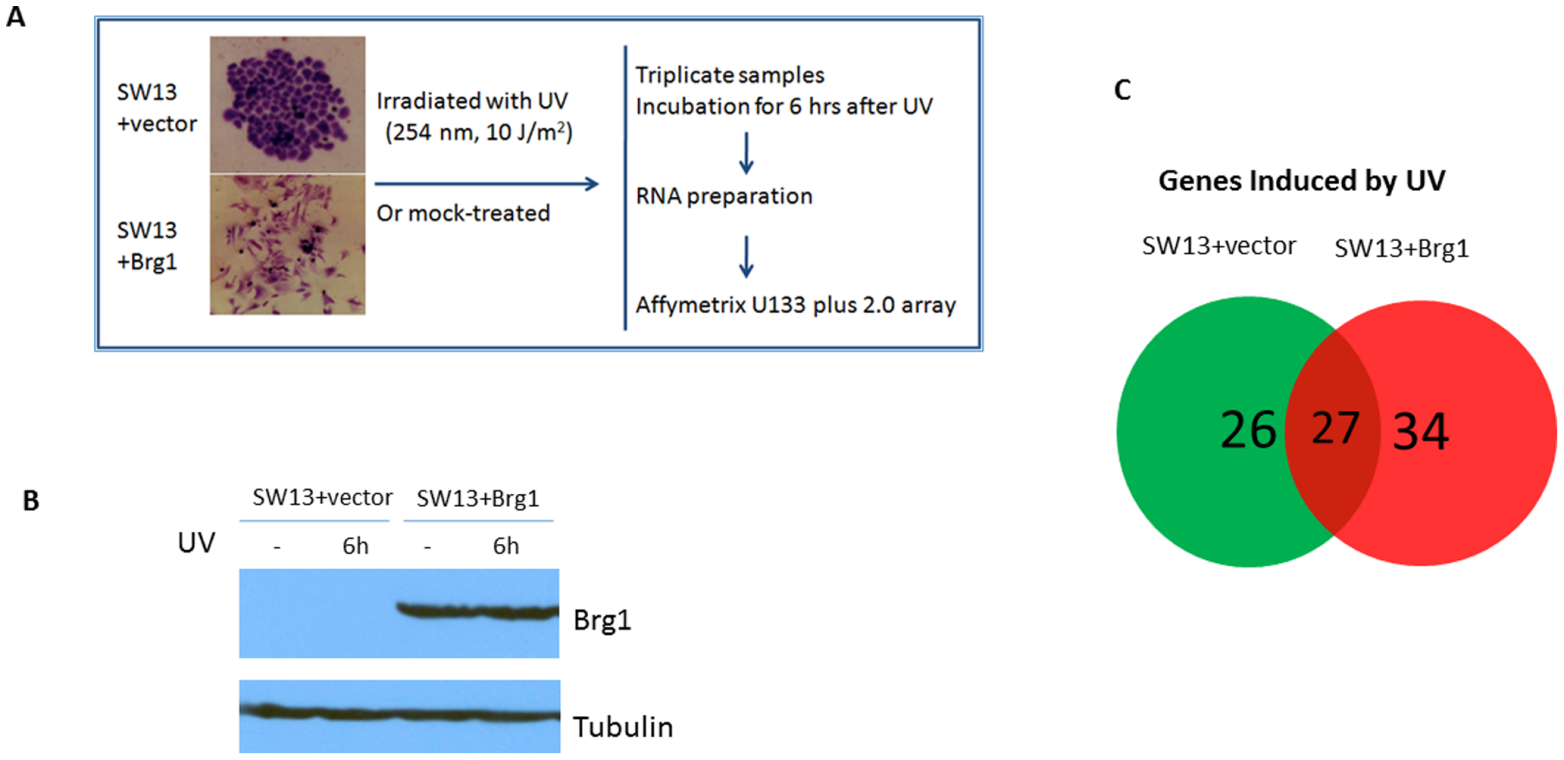
Microarray analysis of gene expression in response to UV irradiation in SW13 cells with or without BRG1. (**A**) Morphological changes of SW13 cells after BRG1 re-expression and a schematic representation of sample preparation for Microarray analysis. SW13 cells with and without reintroduction of BRG1 were irradiated with UV or mock-treated. Three independent biological samples of these treatments were incubated for 6 hours in warm media to allow for recovery, and then collected for RNA extraction. RNA preparations were then used for Microarray analysis. Interestingly, this figure also shows the morphological changes associated with reintroduction of BRG1 in SW13 cells. (**B**) Western blotting confirming BRG1 protein expression. (**C**) Venn Diagram showing that BRG1 regulates the transcriptional response to UV irradiation in human cells. Shown here are the number of genes induced by UV in SW13 cells and SW13+BRG1 cells. 34 of these genes are BRG1-dependent genes, and are not expressed without BRG1 reintroduction. Out of the 87 total genes induced by UV, 61 of them are only induced in cells which include expression of BRG1. Thus, more than 70% of UV induced genes are regulated by BRG1.

As shown in Fig. 1, SW13+pREP7 and SW13+pREP7+BRG1 cells were irradiated with 10 J/m2 UV or mock treated. After treatment, warm media were added back and cells were incubated for 6 hours before being collected for RNA preparation. SW13 cells were treated with 50 µM demethylating agent 5-aza-2′-deoxycytidine (5-Aza) and incubated for 4 days before being collected for RNA extraction.

### Microarray analysis

Total RNA was prepared using TRIZOL reagent (Invitrogen, Carlsbad, CA), followed by the RNeasy kit (Qiagen, Valencia, CA), according to the manufacturer's instructions. Three independent biological replicates of SW13+pREP7 and SW13+pREP7+BRG1 were subjected to microarray analysis using Affymetrix U133 plus 2.0 gene chip. Target synthesis and GeneChip hybridization, washing, staining, and scanning were performed at the Molecular Biology Core at Washington State University. Microarray output was examined visually for excessive background noise and physical anomalies. The default MAS statistical values were used for all analyses. All probe sets on each array were scaled to a mean target signal intensity of 125, with the signal correlating to the amount of transcript in the sample. An absolute analysis using MAS was performed to assess the relative abundance of the 47,000 represented transcripts and variants, including 38,500 human genes, based on signal and detection (present, absent, or marginal). The resulting data from the absolute analysis were exported into Microsoft EXCEL and then imported into GeneSifter software (GeneSifter.net, Seattle, WA). Transcripts expressed differentially at a statistically significant level were determined using the Welch t-test (variances not assumed equal) with a P-value cutoff of 0.05.

### DNA-Chip Analyzer (dChip) analysis

**Figure 2:**
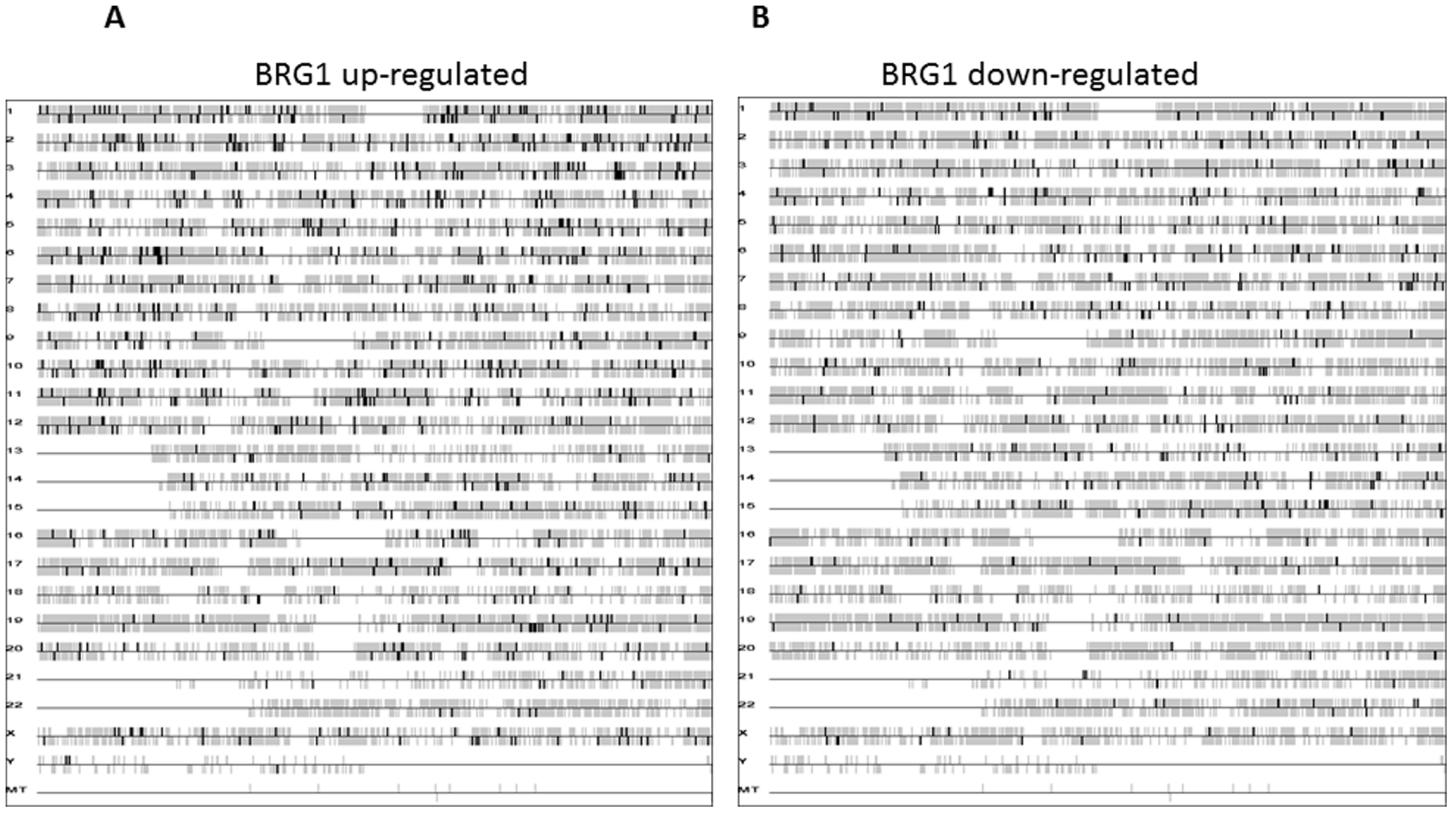
Whole genome view of BRG1 regulatory gene targets (in the absence of UV irradiation) showing the locations of genes on each human chromosome. The vertical bar above the horizontal line indicates genes on the forward strand, and vertical lines below the horizontal line indicate genes on the reverse strand. (A) Genes upregulated by BRG1. (B) Genes downregulated by BRG1.

The bird's eye view of gene location in the genome, as seen in Fig. 2, was generated using dChip, a software package implementing model-based expression analysis of oligonucleotide arrays [31]. The transcription starting site is used for gene position. The vertical bar above the horizontal line means that the gene is on the forward strand, and vice versa.

### Reverse transcription-PCR (RT-PCR) and quantitative real time PCR

Total RNA samples generated for the microarray experiment as described above, were also used for RT-PCR after DNase I digestion (Ambion, Austin, TX). Single-strand cDNA was synthesized using the Reverse Transcription System (Promega, Madison, WI) using oligo(dT)_12–18_ as primer. PCR was performed subsequently using RedTaq DNA polymerase (Sigma, St. Louis, MO) as described with primers listed in Table S1A. The PCR conditions were: 94°C, 30 sec; 52°C, 30 sec; and 72°C, 1 min, for 35 cycles. The cDNA was used as template in a quantitative real-time PCR using a MJ Mini Personal Thermal Cycler (Biorad). Reactions were done in a final volume of 20 µl in SsoFast EvaGreen Supermix (Biorad). Primer sequences are listed in the Table S1A. The mRNA expression levels for all samples was normalized to the levels of GAPDH.

### Chromatin immunoprecipitation assays

Chromatin immunoprecipitation assays (ChIP) were performed using the protocol described on Upstate Cell Signaling website (http://www.millipore.com/techpublications/tech1/mcproto407) in 293T cell line. Immunoprecipitations were done using 4ug of the rabbit anti-BRG1 antibody (H-88) from Santa Cruz Biotechnology. The primers used to amplify *E*-*cadherin, PML*, *RGC32/c13 orf15*, *EGFR*, *S100A2*, *TNIK*, *ARHGAP29/PARG1*, and *ATF3* from the promoter region are listed in Table S1B. The PCR conditions were similar to those described above.

## Results and Discussion

DNA repair and transcriptional activation are two important cellular responses to deal with UV damage [32]. Since BRG1, an ATPase of the SWI/SNF-like BAF complexes, is a major co-regulator of transcription in mammalian cells [33], this study sought to examine its role in protecting human cells against UV radiation. To study the expression profile of genes in response to UV and mock treatment, we used isogenic cell lines, SW13+pREP7 (SW13+vector) and SW13+pREP7+BRG1 (Fig. 1A). BRG1 expression was confirmed by Western blot analysis and it is apparent that UV treatment did not affect BRG1 expression in SW13+Brg1 cells (Fig. 1B). By comparison with earlier studies, our investigation using oligonucleotide microarray technology (Affymetrix Human Genome U133 Plus 2.0 GeneChips) provides a more comprehensive profile of gene expression. We found that BRG1 regulates more than 70% of UV inducible genes, which are dispersed throughout the genome (Fig. 1C), some of which have been shown to be involved in UV damage repair and signaling. Surprisingly, by comparing gene expression in SW13+pREP7 and SW13+pREP7+BRG1 cells in the absence of UV treatment, this study shows that BRG1 regulates gene transcription in whole human genome, in a global fashion (Fig. 2). We show that altogether, about 4.8% of the human genome is transcriptionally regulated by BRG1, which is comparable with previous findings that the SWI/SNF complex regulates 6% of genes in yeast [34]. Thus, our study establishes that BRG1 availability in mammalian cells affects cellular transcriptional response to UV radiation. In addition, based on the genome expression data, our ChIP studies identified several novel transcriptional targets directly regulated by BRG1, warranting future experiments to fully understand BRG1's role as a tumor suppressor.

### BRG1 transcriptionally regulates stress response genes that are induced by UV radiation

Transcriptional response is one of several ways cells deal with stress associated with DNA damage. Other responses include activation of DNA repair pathways and cell cycle checkpoints to arrest cell cycle progression and apoptosis [32]. Previously, we have shown that BRG1 suppresses UV induced apoptosis in SW13 cells [35], but the underlying mechanisms remain undefined. Gene expression profiling showed that UV radiation significantly (>2 fold induction, p<0.05) induces expression of 53 genes in SW13+pREP7 cells, 61 genes in SW13+pREP7+BRG1 cells (Tables S2A and S2B, respectively). Interestingly, 34 genes induced by UV are BRG1-dependent (Fig. 1C and Table 1). Many of those UV-inducible genes are transcription factors, which directly enhance/repress the transcription of their target genes, thus regulating their ability to exert biological functions in cellular responses to protect cells again the toxic effect of UV radiation. Also among these BRG1-dependent UV inducible genes, *ATF3*, *Gadd45a* and *p21* are known to play important roles in cellular resistance to UV radiation [36]–[38], and our microarray studies show that they exhibit higher levels of expression in SW13 cells when BRG1 is re-introduced. These data confirm our previous RT-PCR observations that BRG1 regulates expression of p21 and Gadd45a [35], and that BRG1 binds to *Gadd45a* and *p21* promoter regions to regulate their expression [12], [39], [40].

**Table 1:** 34 Genes Induced by UV Radiation in a BRG1-Dependent Manner.

Probe Set ID	Gene Symbol	Gene Title
202284_s_at	CDKN1A	Cyclin-dependent kinase inhibitor 1A (p21, Cip1)
202672_s_at	ATF3	Activating transcription factor 3
202708_s_at	HIS2H2BE	Histone cluster 2, H2be
202859_x_at	IL8	Interleukin 8
203725_at	GADD45a	Growth arrest and DNA inducible, alpha
204621_s_at	NR4A2	Nuclear receptor subfamily 4, group A, member 2
205193_at	MAFF	v-maf musculoaponeurotic fibrosarcoma oncogene homolog F (avian)
207064_s_at	AOC2	Amine oxidase, copper containing 2 (retina-specific)
211506_s_at	IL8	Interleukin 8
214169_at	UNC84A	Unc-84 homolog A (*C. elegans*)
220533_at		Transcribed locus
36711_at	MAFF	v-maf musculoaponeurotic fibrosarcoma oncogene homolog F
38037_at	HBEGF	Heparin-binding EGF-like growth factor
223218_s_at	NFKBIZ	Nuclear factor of kappa light polypeptide gene enhancer in B-cells inhibitor, zeta
228839_s_at	LOC642361	Hypothetical gene supported by AF064843; AK025716 /// hypothetical LOC642361
230604_at		Transcribed locus
231233_at		Transcribed locus
231417_at		Transcribed locus
232097_at	TOX4	TOX high mobility group box family member 4
237116_at	LOC646903	Hypothetical LOC646903
238012_at	DPP7	Dipeptidyl-peptidase 7
238633_at	EPC1	Enhancer of polycomb homolog 1 (*Drosophila*)
239814_at		Transcribed locus, strongly similar to XP_531062.1 PREDICTED: hypothetical protein [Pan troglodytes]
242255_at	LOC100130837	Hypothetical protein LOC 100130837
242594_at	FAM44A	Family with sequence similarity 44, member 4
243404_at		Transcribed locus
243947_s_at		Transcribed locus
1552362_a_at	LEAP2	Liver expressed antimicrobial peptide 2
1554020_at	BICD1	Bicaudal D homolog 1 (*Drosophila*)
1554980_a_at	ATF3	Activating transcription factor 3
1556213_a_at	BTG3	BTG family, member 3
1556216_s_at		cDNA clone IMAGE: 5261375
1556346_at		Partial mRNA; ID YG39-1A
1556588_at	C15orf37	Chromosome 15 open reading frame 37
1557104_at	ZNF397OS	Zinc finger protein 397 opposite strand
1560129_at		mRNA; cDNA DKFZp313H0240 (from clone DKFZp313H0240)
1565759_at	RPL 13	Ribosomal protein L 13

**Figure 3:**
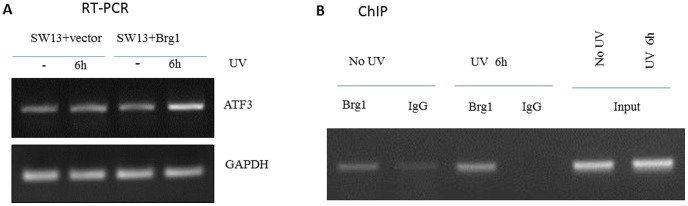
BRG1 controls ATF3 expression under UV conditions. (**A**) RT-PCR analysis of ATF3 gene expression in SW13+vector and SW13+BRG1 cells. Both cells were collected 6 hours after UV treatment. RNA was purified and RT-PCR products were analyzed on agarose gel. (**B**) Binding of BRG1 to ATF3 gene promoter region. 293T cells were UV irradiated and incubated for 6 hours followed by Chromatin Immunoprecipitation (ChIP) assay with anti-BRG1 antibody.

Although it has been shown that ATF3 acts cooperatively with BRG1 to activate promoters, and plays a role in recruitment of BRG1 to gene promoters [41], this study identifies for the first time that under UV induction, BRG1 controls *ATF3* expression. ATF3, a transcription factor in the ATF/CREB family, plays a significant role in cells stress response. ATF3 protein is expressed at a low levels in normal and quiescent cells, but can be rapidly and highly induced in response to multiple and diverse extracellular signals [42] including UV irradiation [43]. Importantly, overexpression of ATF3 protein suppresses cell growth [44]. Interestingly, BRG1 binds to the *ATF3* promoter both, with and without UV irradiation, but binds better under UV conditions and higher expression of *ATF3* is seen under these conditions as well (Fig. 3). This seems to suggest that there are perhaps other transcription factors activated by UV that aid BRG1 in binding to the *ATF3* promoter. Further studies are necessary to fully understand this data, but our data newly demonstrate that BRG1 plays a direct role in activating expression of *ATF3* under UV conditions.

Since SW13 cells have shown attenuated repair of CPDs, while BRG1 restoration has been shown to correct this deficiency [12], it might be possible to infer that BRG1 transcriptionally regulates expression of genes involved in NER. From our microarray data, we singled out the expression profile of NER genes. Although BRG1 expression leads to small changes in NER gene expression, none of the changes seem to be significant (>2 fold with a p value <0.05). Therefore, under our experimental conditions, we cannot conclude that BRG1 transcriptionally regulates expression of NER genes.

### BRG1 expression changes global gene expression in the absence of UV radiation

**Figure 4:**
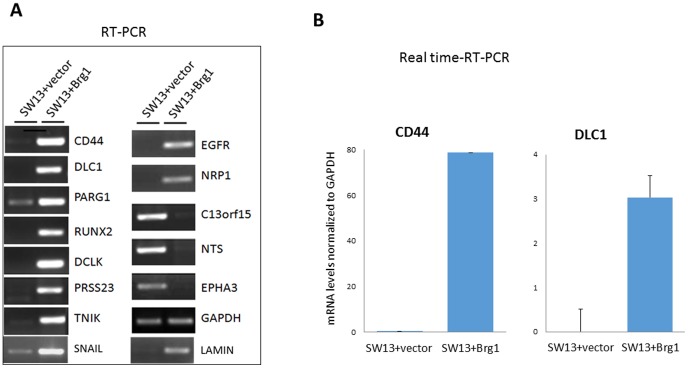
Validation of the Microarray data by RT-PCR. (**A**) Florescence intensity of RT-PCR products as a measure of mRNA expression confirming microarray data. Upon reintroduction of BRG1 into SW13 cells, regulation of gene expression differs from those cells without BRG1. GAPDH was loaded as a control. (**B**) Real time RT-PCR quantitation of CD44 and DLC1 mRNA levels.

The design of this study also allowed us to identify novel transcriptional targets of BRG1 in the absence of UV treatment, by querying the global gene expression profile of the SW13+pREP7+BRG1 cells in comparison with SW13+pREP7 cells. Based on comparison of the SW13+vector vs. SW13+pREP7+BRG1 transcript abundance ratio, a value of >2.00 identified 1004 genes with reduced transcript levels and 599 genes with increased transcript levels in the SW13+pREP7+BRG1 (Tables 2 and S3A). Among these, *CD44* [45], *Gadd45* [30], [46] and *p21* [39], [46] have previously been shown to be up-regulated by BRG1 in SW13 cells, validating our system for the study of gene regulation by BRG1. RT-PCR analysis of several highly up-regulated and down-regulated genes (*CD44*, *DLC1*, *PARG1*, *RUNX2*, *DCLK*, *PRSS23*, *TNIK*, *EGFR*, *NRP1*, *C13orf15*, *NTS*, *EPHA3*, *SNAIL*, *LAMIN*, and *GAPDH*), was performed to confirm the microarray data, and have shown to be consistent with the microarray data (Fig. 4A). Real time RT-PCR quantitation again confirms that BRG1 up-regulates expression of *CD44* and *DLC1* (Fig. 4B).

**Table 2:** Comparison of BRG1 regulated gene expression with UV treatment and without.

	No UV	UV 6 hours	Overlapping genes
**Up-regulated genes**	599	806	62
**Down-regulated genes**	1004	1249	108

### Identification of novel BRG1 transcriptional targets

**Figure 5:**
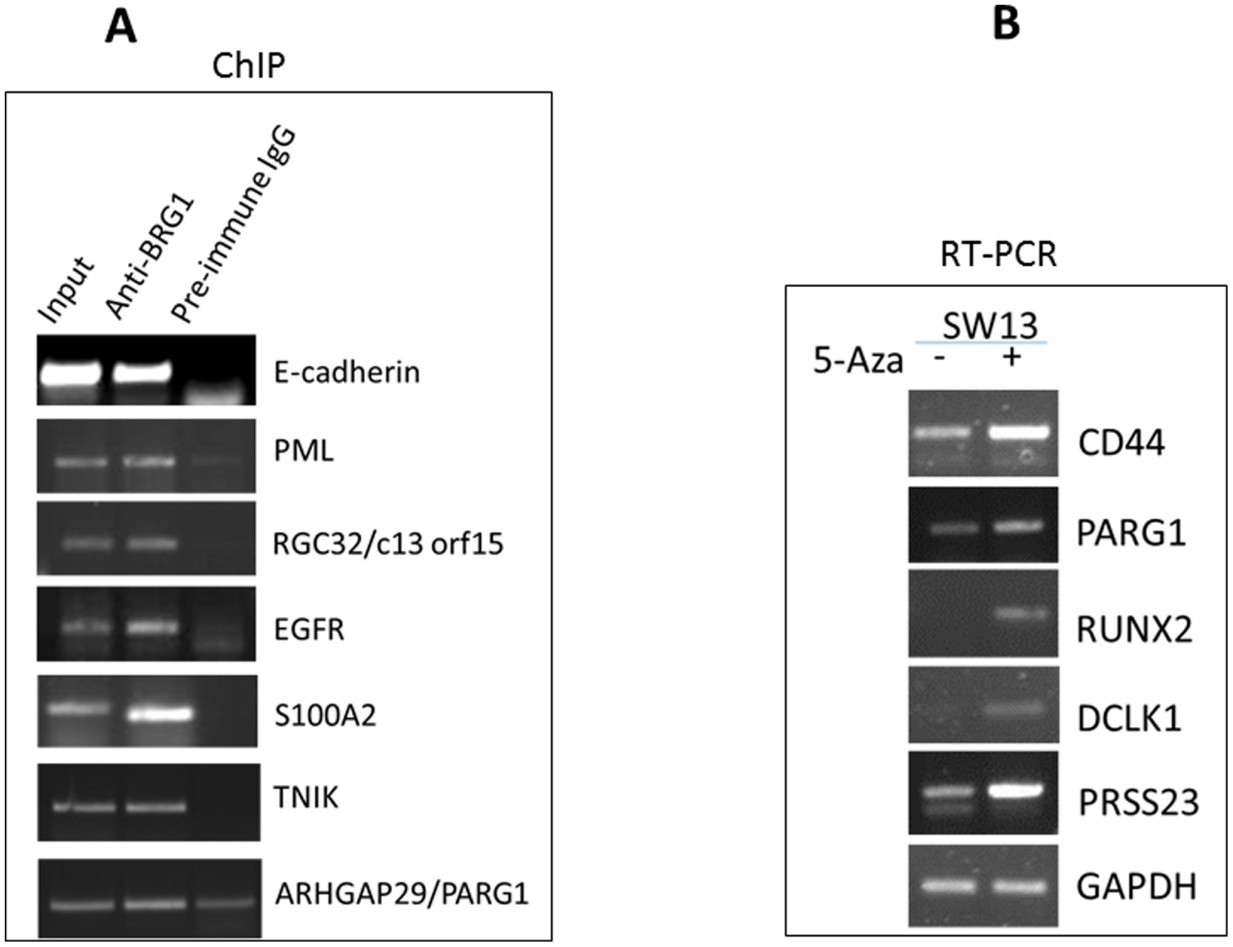
Validation of the Microarray data by RT-PCR and ChIP. (**A**) Chromatin Immunoprecipitation studies show that BRG1 binds to the promoter of these genes, and regulates their expression. (**B**) RT-PCR showing that treatments with 5-aza-2′-deoxycytidine (5-Aza) induces expression of some of the identified BRG1 target genes in SW13 cells, which lack endogenous BRG1 expression.

This study identifies many novel BRG1 transcriptional targets, including *ATF3*, *TNIK*, *PARG,* and *S100A2*, that may help explain the roles of BRG1 in DNA damage response. To determine if SWI/SNF complex containing BRG1 interacts directly with its target gene promoter, chromatin immunoprecipitation (ChIP) assays were performed and several of the genes found to be up-regulated in the global expression study were evaluated. Interestingly, eight of these genes exhibited binding of BRG1 to their promoters: *E-cadherin*, *PML*, *RGC32/c13 orf15*, *EGFR*, *S1002A*, *TNIK*, *ARHGAP29/PARG1*, and *ATF3*. As shown in Fig. 5A, the promoter sequence was significantly enriched in the immunoprecipitates with anti-BRG1 antibody in 293T cell lines. This study indicates that BRG1 has a direct role in transcriptional regulation of several of its target genes, and likely more of them, as well.

It has been previously shown that the promoters of *E-cadherin* and *CD44* are hypermethylated when BRG1 (or BRM) is absent from cells, and that BRG1 associates with the CpG islands in the promoter regions of the genes [47]. These authors proposed that the loss of SWI/SNF-mediated transcriptional activation increases DNA methylation in cancer cells. After confirming BRG1's association with promoters of several genes, (Fig. 5A), we performed demethylating studies with these genes, using 5-aza-2′deoxycytidine (as previously done, [47]) in SW13 cells that lack BRG1 expression. Similar to the findings in the previous studies, we also found induced expression of these genes upon demethylation treatment (Fig. 5B). This, in correlation with our microarray data and our RT-PCR data (Fig. 4) which show that BRG1 regulates expression of *E-cadherin* and *CD44*, led us to hypothesize that perhaps BRG1 regulates expression of these genes through the same mechanism proposed previously: by demethylating sequences in the promoter regions.

### BRG1 is involved with regulation of many pathways

Our study shows that approximately 4.8% of the human genes are transcriptionally regulated by BRG1. Interestingly, analysis of our microarray data shows that BRG1 does not regulate genes within localized ‘hot-spots’, but rather plays a regulatory role of transcription throughout the whole genome, as seen in Fig. 2. Indeed, functional classification of the BRG1 responsive genes into different pathways (Table 3) indicates that BRG1 regulates various cellular pathways such as, MAPK signaling, cell cycle, regulation of actin cytoskeleton, cell adhesion, apoptosis, GTPase-mediated signal transduction, and transcriptional regulation. BRG1 does not regulate an exclusive signaling pathway; instead, multiple cellular pathways are affected upon re-expression of BRG1 in SW13 cells. Thus, this study highlights the notion that BRG1/BAF complexes serves as a fundamental component of various pathways.

**Table 3:** BRG1 regulates multiple cellular pathways.

KEGG Pathway	Total Genes Regulated by BRG1	Up-regulated	Down-regulated
MAPK Signaling Pathway	46	29	17
Focal Adhesion	44	35	9
Regulation of actin cytoskeleton	37	25	12
Cytokine-cytokine receptor interaction	33	26	7
Axon guidance	29	15	14
Wnt signaling pathway	29	18	11
Calcium signaling pathway	28	15	13
Neuroactive ligand-receptor interaction	28	18	10
Cell communication	26	17	9
Cell cycle	26	5	21
Pancreatic cancer	18	14	4
Colorectal cancer	16	11	5
Basal cell carcinoma	15	10	5
Melanoma	15	11	4
Glioma	14	11	3
Renal cell carcinoma	14	12	2
Small cell lung cancer	14	11	3
Non-small cell lung cancer	11	10	1
Prostate cancer	11	11	0

Our microarray data indicate that BRG1 plays a role in regulating expression of many genes in the MAPK signaling pathway (Table 3). Deregulation of the MAPK pathways has been widely associated with tumorigenesis and has found to be deregulated in approximately 1/3 of cancers [48]. JNK has been shown to bind to and phosphorylate p53 in response to stresses such as UVB radiation and DNA damage [49]. This interaction with p53 is thought to increase its transcriptional expression and stability [50]. Thus, proper BRG1 expression is important for proper maintenance of cell signaling and p53 activity, through the MAPK pathway.

Activation of cell cycle checkpoint to arrest cell cycle progression and apoptosis are other ways that cells deal with stress associated with DNA damage [32]. Our cDNA microarray data (Table 1) not only agree with previous observations that *BRG1* reintroduction into SW13 cells induces gene expression of p21, p15 and GADD45a [30], but also reveal more BRG1-regulated genes that are involved in cell cycle control, including *p16*, *SMAD3*, *CCNG1*(*Cyclin G1*), *CCNG2* (*Cyclin G2*) and *PML* (*Promyelocytic leukemia*) genes.

Consistent with previous reports [30], our microarray and RT-PCR experiments demonstrate that BRG1 induces expression of several genes involved in cell adhesion and differentiation. Given that reintroduction of *BRG1* induces changes in overall cell shape and morphology [30], [51], [52], and the cells become flatter with altered cytoskeletal organization (Fig. 1), BRG1's regulation of genes involved in cell morphology and development is crucial in understanding how cell function is differentially regulated. As reported in earlier studies [30], [47], our data show upregulation of *CD44* and *E-cadherin* by BRG1 (Table S3B), and that BRG1 binds to the promoters of the genes (Fig. 5A). CD44 associates with moesin, which links the plasma membrane with the actin cytoskeleton. Cadherin-based adherens junction found between polarized epithelial cells, are also intimately associated with the actin cytoskeleton.

This study also shows BRG1 upregulated expression of TNIK and PARG1 (Table S3B and Fig. 4A). TNIK is known to be an essential activator of the Wnt signaling pathway and an integral part of canonical and non-canonical Wnt pathways [53], [54] and PARG1 is a Rho GTPase activating protein. The upregulation of these two genes suggests that BRG1 could control one or both of them for intricate control of actin cytoskeleton. Our ChIP data confirms BRG1 upregulation of most of these genes, as seen by BRG1 binding to their promoters in Fig. 5A. Our microarray and RT-PCR studies (Table S3B and Fig. 4A) also show that BRG1 upregulates *SNAIL* and *LAMIN* genes. SNAIL is a transcription factor that is known to bind to *E-cadherin* promoters and repress its transcription, thus inducing Epithelial-mesenchymal transition (EMT) [55], which is a crucial event in tumor progression. LAMIN is fundamental component of the nuclear lamina, which is involved in cross-talk with several cancer-regulating pathways, and its altered expression has been associated with a wide variety of cancers [56]. High amounts of LAMIN A have been shown to interfere with cell migration [57], a crucial process that occurs in EMT. Thus, proper levels of SNAIL and LAMIN are important for EMT to remain in a healthy balance, and BRG1 may regulate EMT and cancer metastasis through transcriptional regulation of these genes. Indeed, our pathway analysis showed that BRG1 regulates many genes involved in several cancer types (Table 3), and *BRG1* is frequently deleted or mutated in a variety of tumor cell lines, including carcinomas of pancreas, lungs, prostate, breast [22]. BRG1 functions as a tumor suppressor, and regulation of gene expression through chromatin remodeling is critical for tumor progression. The functional balance of BRG1-regulated genes might dictate the likelihood of a given cell becoming cancerous or not.

### BRG1 regulates a significant number of p53 targets

SWI/SNF-like BAF complexes have been shown to physically interact with p53 [58], [59], suggesting that p53 may recruit BAF complexes to its targeted promoters, and that the SWI/SNF complex may function as a tumor suppressor via interactions with p53. In fact, in vivo studies have shown BRG1 recruitment to p53-dependent promoters [59]. Therefore, we examined if BRG1 affects transcriptional expression of p53 target genes. A comparison of BRG1 transcriptional targets and reported p53 targets (Table 4) reveals that a significant number of p53-regulated genes are also regulated by BRG1, such as *ATF3*, *PML*, *p21*, *Gadd45a*. ATF3 has been shown to bind to p53 and prevent its ubiquitination upon DNA damage, thereby stabilizing p53 and enhancing its transcriptional activity and tumor suppressor function [60]. Recently, ATF3 has also been implicated as a crucial co-transcription factor for p53 upon DNA damage [36]. PML is well-known to form complexes with p53 upon UV radiation, thus activating and stabilizing p53 for further tumor suppressor action [61]. Another overlapping gene target, p21, is well-known to be recruited by p53 to arrest cell growth in the event of DNA damage [37]. In addition, Gadd45a, a ubiquitously expressed protein involved in growth arrest and apoptosis induced by UV damage, is highly intertwined with the p53 pathway, helping to activate it, which at the same time being regulated by it [38]. These overlapping gene targets suggest that p53 may require BRG1 to regulate its downstream targets. Thus, our findings support the notion that the tumor suppressor role of BRG1 is mediated in part through the p53 pathway [62].

**Table 4:** List of BRG1 regulated p53 targets.

Gene Name	GenBank ID	Affimetrix	Gene Description	References
CAV1	NM_001753	203065_s_at	Caveolin 1, caveolae protein, 22kDa	[65]
MET	BG170541	203510_at	Met proto-oncogene (hepatocyte growth factor receptor)	[66]
IFI16	NM_005531	206332_s_at	Interferon, gamma-inducible protein 16	[67]
IGFBP3	M31159	210095_s_at	ATP-binding cassette, sub-family C (CTFR/MRP), member 3	[68]
S100A2	NM_005978	204268_at	S100 calcium binding protein A2	[69]
PMAIP1	AI857639	204285_s_at	Phorbol-12-myristate-13-acetate-induced protein 1	[70]
TGFA	M31172	205015_s_at	Transforming growth factor, alpha	[71]
MVP	NM_017458	202180_s_at	Major vault protein	[72], [73]
IER3	NM_003897	201631_s_at	Immediate early response 3	[74]
GADD45a	NM_001924	203725_at	Growth arrest and DNA damage-inducible, alpha	[75]
EGFR	NM_005228	201984_s_at	Epidermal growth factor receptor (erythroblastic leukemia viral (v-erb-b) oncogene homolog, avian)	[76]
LIF	NM_002309	205266_at	Leukemia inhibitory factor (cholinergic differentiation factor)	[77]
PML	NM_002675	206503_x_at	Promyelocytic leukemia	[78]
BNIP3L	ALI32665	221478_at	BCL2/adenovirus E1B 19kDa interaction protein 3-like	[79]
IL15	Y09908	217371_s_at	*H. sapiens* mRNA for interleukin 15/PROD = interleukin 15	[80]
STEAP3	NM_018234	218424_s_at	STEAP family member 3	[81]
KRT8	U76549	209008_x_at	Keratin 8	[82]
SCARA3	NM_016240	219416_at	Scavenger receptor class A, member 3	[83]
SYKQ	NM_003177	207540_s_at	Spleen tyrosine kinase	[84]
CCNG1	BC000196	208796_s_at	Cyclin G1	[85]
DUSP5	U16996	209457_at	Dual specificity phosphatase 5	[86]
NINJ1	NM_004148	203045_at	Ninjurin 1	[87]
DKK1	NM_012242	204602_at	Dikkopf homolog 1 (*Xenopus laevis*)	[88]
EGR1	AI459194	227404_s_at	Early growth response 1	[89]
TP73L	AB010153	211194_s_at	Tumor protein p73 like	[90]
ARID3A	NM_005224	205865_at	AT rich interactive domain 3A (BRIGHT-like)	[81]
CHEK1	NM_001274	205394_at	CHK1 checkpoint homolog (*S. pombe*)	[91]

We note that the identified BRG1 transcriptional targets in this study may include genes regulated by another BAF complex ATPase, BRM, since BRG1 and BRM have partial functional redundancy [20], [45], [63], [64]. Additional studies will be needed to dissect the redundant and non-redundant roles of the individual ATPase subunits in gene regulation.

In conclusion, this study establishes that transcriptional response to UV damage is regulated by BRG1 in human cells. Several BRG1 up-regulated genes, including *ATF3*, *EGFR*, *p21*, and *Gadd45a*, are all involved in cellular defense against UV radiation [36]–[38]. Thus, it appears that the mechanisms by which BRG1 suppresses UV induced cell death in SW13 cells are multifaceted, playing roles in several different cell stress response pathways. Finally, our understanding of the mechanistic basis of diverse cellular processes, including cell cycle and actin cytoskeleton regulation, has been augmented by the transcriptional profiling of the BRG1 gene. There is abundant evidence that the mechanistic basis of BRG1 function is determined by the transactivation and transrepression of target gene expression [33], [41]. Our study identifies several important new transcriptional targets regulated by BRG1 and a large amount of possible new gene targets. The experiments presented here will serve as a platform for investigating the direct role of BRG1 in gene regulation, and will contribute toward the development of a wide range of intelligent hypotheses and drive a new generation of functional experiments.

## Supporting Information

Table S1 **PCR primers used in this study.**
(DOCX)

Table S2 **Table S2A: List of genes induced by UV in SW13+vector cells.** **Table S2B:** List of genes induced by UV in SW13+Brg1 cells.
(DOCX)

Table S3 **Table S3A: Genes up- or down-regulated by BRG1 in the absence of UV irradiation. Table S3B**: Genes up- or down-regulated by BRG1 6 hours after UV irradiation.
(XLSX)
