# Supplementary material for: Whole Genome Expression Profiling Shows that BRG1 Transcriptionally Regulates UV Inducible Genes and Other Novel Targets in Human Cells
Source: PLoS One. 2014 Aug 26;9(8):e105764. doi: 10.1371/journal.pone.0105764 (PMC4144907; doi:10.1371/journal.pone.0105764)
Supplement: Table S1 — PCR primers used in this study. (DOCX) [file pone.0105764.s001.docx]

**Table S1A. Primers used in ChIP study**

| Gene Name | Primers |
| --- | --- |
| *E-cadherin* | Forward 5’-GAACTCAGCCAAGTGTAAAAGCC-3’  Reverse 5’-GAGTCTGAACTGACTTCCGC-3’ |
| *PML* | Forward 5’-GAGCTAAAGGCATGTCCCAGA-3’  Reverse 5’-GCCTGGAACTTGTCATCCTCC-3’ |
| *RGC32/c13 orf15* | Forward 5’-TCTTCCCATAATAAACCCC-3’  Reverse 5’-TCCCCTACGCATTGTCCCAA-3’ |
| *EGFR* | Forward 5’-ACATTAAGGAGGCCTGTCT-3’  Reverse 5’-AGCAAACTTGTACCAGCTT-3’ |
| *S100A2* | Forward 5’-TGTCATGAGAATGAGGCCT-3’  Reverse 5’-AAACCTGCCTCAACCTGAT-3’ |
| *TNIK* | Forward 5’-GTGCGTGTGCGCTTCTTCC-3’  Reverse 5’-CCTTGACTTGTTATCATAGG-3’ |
| *ARHGAP29/PARG1* | Forward 5’-GATCCACAGAAGTGTCTCA-3’  Reverse 5’-AGGTACGGGAGGCAACTA-3’ |
| *ATF3* | Forward 5’-AACCTAGCGGAGGGACAGAT-3’  Reverse 5’-CTCTCTCCATATCAGGACC-3’ |

**Table S1B. Primers used in RT-PCR study**

| Gene Name | Primers |
| --- | --- |
| *DLC1* | Forward 5’-CACAGGACAACCGTTGCCTCAG-3’  Reverse 5’-CTCTTCAGGGTGTTGAGATGGA-3’ |
| *PARG1* | Forward 5’-GATAGTTTTTCACCCCTTG-3’  Reverse 5’-AACCCAGAAAATAACAGGA-3’ |
| *RUNX2* | Forward 5’-ACACTTCTTCCAAACCTTG-3’  Reverse 5’-ACAATCATGACCTGTGGCA-3’ |
| *CD44* | Forward 5’-AATCTTTTGTGTCTCCTGA-3’  Reverse 5’-GGTGTGCTCTGAAAAGTCAA-3’ |
| *TNIK* | Forward 5’-ATATGCTTGGGCTCCTAAAC-3’  Reverse 5’-AATGAATGTAGGCCACAGAC-3’ |
| *EGFR* | Forward 5’-GTCAACAGCACATTCGACA-3’  Reverse 5’-CTTCCCAAATGTGCCCGA-3’ |
| *DCLK1* | Forward 5’-GGTTACATACTTTCTGCT-3’  Reverse 5’-TAGTTATTCTAAGGTGGT-3’ |
| *PRSS23* | Forward 5’-AGACTGTCAGTACTGGGAG-3’  Reverse 5’-CTTAGGTCCCTGATGCGGCCAA-3’ |
| *NRP1* | Forward 5’-AATATCTTACCAGGCAGCC-3’  Reverse 5’-CCTTGGCTGAGGACAACTTT-3’ |
| *C13orf15* | Forward 5’-CTGAATTCTCCAACAGACT-3’  Reverse 5’-ATGGGAAAGCTTACTGCT-3’ |
| *NTS* | Forward 5’-CCCTCTTGGAAGATGACTC-3’  Reverse 5’-GATGAAGAGACAGATAAGTG-3’ |
| *EPHA3* | Forward 5’-GCATACTCAACAAAACCCATG-3’  Reverse 5’-GAGTGAGTTACCAGAAATAA-3’ |
| *GAPDH* | Forward 5’-GAAGGTGAAGGTCGGAGTCA-3’  Reverse 5’-GAAGATGGTGATGGGATTTC-3’ |
| *SNAIL* | Forward 5’- GTATCCAGAGCTGTTTGGA-3’  Reverse 5’-AACATTTTCCTCCCAGGCC-3’ |
| *LAMIN* | Forward 5’-AAAATGGAGATGCCTAGCA-3’  Reverse 5’-TGGGACGCGTTGAACAAGG-3’ |
