# Supplementary material for: Whole Genome Expression Profiling Shows that BRG1 Transcriptionally Regulates UV Inducible Genes and Other Novel Targets in Human Cells
Source: PLoS One. 2014 Aug 26;9(8):e105764. doi: 10.1371/journal.pone.0105764 (PMC4144907; doi:10.1371/journal.pone.0105764)
Supplement: Table S2 — A: List of genes induced by UV in SW13+vector cells. Table S2B: List of genes induced by UV in SW13+Brg1 cells. (DOCX) [file pone.0105764.s002.docx]

**Table S2A**. List of genes induced by UV in SW13+vector cells.

| **Probe Set ID** | **Fold change** | | **Unigene(Avadis)** | | **Gene Symbol** | **Gene Title** | | | | |  |  |  |  |  |  |  |  |
| --- | --- | --- | --- | --- | --- | --- | --- | --- | --- | --- | --- | --- | --- | --- | --- | --- | --- | --- |
| 202708_s_at | 2.617554 | Hs.2178 | | HIST2H2BE | | | histone cluster 2, H2be | | |  | |  |  |  |  |  |  |  |
| 202814_s_at | 2.045586 | Hs.15299 | | HEXIM1 | | | hexamethylene bis-acetamide inducible 1 | | | | | |  |  |  |  |  |  |
| 202815_s_at | 4.291525 | Hs.15299 | | HEXIM1 | | | hexamethylene bis-acetamide inducible 1 | | | | | |  |  |  |  |  |  |
| 203455_s_at | 3.679646 | Hs.28491 | | SAT1 | | | spermidine/spermine N1-acetyltransferase 1 | | | | | |  |  |  |  |  |  |
| 203821_at | 2.230415 | Hs.799 | | HBEGF | | | heparin-binding EGF-like growth factor | | | | |  |  |  |  |  |  |  |
| 207623_at | 3.608285 | Hs.654958 | | ABCF2 | | | ATP-binding cassette, sub-family F (GCN20), member 2 | | | | | | |  |  |  |  |  |
| 208576_s_at | 5.894797 | Hs.532144 | | HIST1H2BN | | | histone cluster 1, H2bn /// histone cluster 1, H3a /// histone cluster 1, H3d | | | | | | | | | | | |
| 209007_s_at | 2.224767 | Hs.259412 | | C1orf63 | | | chromosome 1 open reading frame 63 | | | | |  |  |  |  |  |  |  |
| 209189_at | 2.52902 | Hs.25647 | | FOS | | | v-fos FBJ murine osteosarcoma viral oncogene homolog | | | | | | |  |  |  |  |  |
| 210592_s_at | 3.80974 | Hs.28491 | | SAT1 | | | spermidine/spermine N1-acetyltransferase 1 | | | | | |  |  |  |  |  |  |
| 212019_at | 2.733843 | Hs.401842 | | RSL1D1 | | | ribosomal L1 domain containing 1 | | | | |  |  |  |  |  |  |  |
| 213750_at | 2.647656 | Hs.401842 | | RSL1D1 | | | ribosomal L1 domain containing 1 | | | | |  |  |  |  |  |  |  |
| 213988_s_at | 2.788825 | Hs.28491 | | SAT1 | | | spermidine/spermine N1-acetyltransferase 1 | | | | | |  |  |  |  |  |  |
| 214481_at | 3.227024 | Hs.134999 | | HIST1H2AM | | | histone cluster 1, H2am | | |  | |  |  |  |  |  |  |  |
| 214696_at | 3.522648 | Hs.597755 | | C17orf91 | | | chromosome 17 open reading frame 91 | | | | |  |  |  |  |  |  |  |
| 219596_at | 3.421259 | Hs.591123 | | THAP10 | | | THAP domain containing 10 | | |  | |  |  |  |  |  |  |  |
| 220167_s_at | 2.941399 | Hs.513537 | | TP53TG3 | | | TP53TG3 protein /// similar to TP53TG3 protein | | | | | |  |  |  |  |  |  |
| 220936_s_at | 4.173585 | Hs.524280 | | H2AFJ | | | H2A histone family, member J | | |  | |  |  |  |  |  |  |  |
| 223666_at | 3.102762 | Hs.316890 | | SNX5 | | | sorting nexin 5 | |  |  | |  |  |  |  |  |  |  |
| 224860_at | 2.138956 | Hs.7517 | | C9orf123 | | | chromosome 9 open reading frame 123 | | | | |  |  |  |  |  |  |  |
| 227943_at | 2.212348 |  | |  | | | Transcribed locus |  |  |  | |  |  |  |  |  |  |  |
| 228213_at | 3.343489 | Hs.524280 | | H2AFJ | | | H2A histone family, member J | | |  | |  |  |  |  |  |  |  |
| 228458_at | 2.099017 | Hs.571098 | | LOC441150 | | | similar to RIKEN cDNA 2310039H08 | | | | |  |  |  |  |  |  |  |
| 228967_at | 4.215072 | Hs.150580 | | EIF1 | | | Eukaryotic translation initiation factor 1 | | | | |  |  |  |  |  |  |  |
| 230136_at | 2.318907 |  | | LOC400099 | | | hypothetical gene supported by BC024195 | | | | | |  |  |  |  |  |  |
| 230304_at | 2.586602 | Hs.143408 | | | | | CDNA clone IMAGE:30332316 | | |  | |  |  |  |  |  |  |  |
| 230380_at | 3.158684 | Hs.245798 | | THAP2 | | | THAP domain containing, apoptosis associated protein 2 | | | | | | |  |  |  |  |  |
| 230393_at | 2.183327 | Hs.440320 | | CUL5 | | | cullin 5 |  |  |  | |  |  |  |  |  |  |  |
| 233376_at | 2.731199 | Hs.661713 | | | | | G protein interaction factor 2-like mRNA sequence | | | | | |  |  |  |  |  |  |
| 235174_s_at | 3.69871 | Hs.708094 | | LOC100128822 | | | hypothetical protein LOC100128822 | | | | |  |  |  |  |  |  |  |
| 235587_at | 2.89677 | Hs.193172 | | LOC202781 | | | hypothetical protein LOC202781 | | | | |  |  |  |  |  |  |  |
| 235607_at | 2.396018 | Hs.660065 | | | | | Transcribed locus | |  |  | |  |  |  |  |  |  |  |
| 236124_at | 3.282434 | Hs.596513 | | LOC153546 | | | hypothetical protein LOC153546 | | | | |  |  |  |  |  |  |  |
| 236213_at | 5.1124 | Hs.666143 | | LOC100130885 | | | hypothetical protein LOC100130885 | | | | |  |  |  |  |  |  |  |
| 236282_at | 2.118908 | Hs.633960 | | | | | CDNA clone IMAGE:4826240 | | |  | |  |  |  |  |  |  |  |
| 236313_at | 2.304686 | Hs.72901 | | CDKN2B | | | cyclin-dependent kinase inhibitor 2B (p15, inhibits CDK4) | | | | | | |  |  |  |  |  |
| 236875_at | 3.249431 | Hs.648517 | | | | | Transcribed locus | |  |  | |  |  |  |  |  |  |  |
| 236898_at | 2.293764 | Hs.550924 | | | | | Transcribed locus, strongly similar to XP_001101634.1 PREDICTED: similar to tripartite motif-containing 25 [Macaca mulatta] | | | | | | | | | | | |
| 237510_at | 3.995537 |  | |  | | | Transcribed locus |  |  |  | |  |  |  |  |  |  |  |
| 238228_at | 2.979091 |  | |  | | | Transcribed locus |  |  |  | |  |  |  |  |  |  |  |
| 238389_s_at | 2.515472 | Hs.222218 | | | | | Transcribed locus, moderately similar to XP_001093747.1 PREDICTED: tumor protein p53 binding protein, 2 [Macaca mulatta] | | | | | | | | | | | |
| 238646_at | 2.443387 | Hs.597327 | | | | | Transcribed locus | |  |  | |  |  |  |  |  |  |  |
| 238813_at | 4.347537 | Hs.555936 | | | | | Transcribed locus | |  |  | |  |  |  |  |  |  |  |
| 238824_at | 2.536847 | Hs.634964 | | | | | CDNA FLJ30581 fis, clone BRAWH2007069 | | | | |  |  |  |  |  |  |  |
| 238946_at | 3.529036 | Hs.665649 | | | | | Transcribed locus | |  |  | |  |  |  |  |  |  |  |
| 239266_at | 2.871572 | Hs.245367 | | | | | Transcribed locus | |  |  | |  |  |  |  |  |  |  |
| 239536_at | 3.610025 | Hs.662791 | | | | | Transcribed locus | |  |  | |  |  |  |  |  |  |  |
| 240324_at | 2.111403 |  | |  | | | Transcribed locus |  |  |  | |  |  |  |  |  |  |  |
| 241471_at | 2.915397 |  | | LOC730236 | | | hypothetical LOC730236 | | |  | |  |  |  |  |  |  |  |
| 241721_at | 3.300943 | Hs.555274 | | | | | CDNA FLJ37844 fis, clone BRSSN2012622 | | | | |  |  |  |  |  |  |  |
| 244397_at | 2.222186 |  | |  | | | Transcribed locus |  |  |  | |  |  |  |  |  |  |  |
| 244786_at | 3.385314 | Hs.448753 | | SNHG10 | | | small nucleolar RNA host gene (non-protein coding) 10 | | | | | | |  |  |  |  |  |
| 1555865_at | 2.281934 | Hs.588291 | | LOC255512 | | | hypothetical protein LOC255512 | | | | |  |  |  |  |  |  |  |
| 1558256_at | 5.463366 | Hs.565253 | | LOC148189 | | | hypothetical protein LOC148189 | | | | |  |  |  |  |  |  |  |
| 1560071_a_at | 3.868638 |  | |  | | | Transcribed locus |  |  |  | |  |  |  |  |  |  |  |
| 1565830_at | 3.303447 | Hs.651430 | | | | | MRNA; cDNA DKFZp686D0673 (from clone DKFZp686D0673) | | | | | | |  |  |  |  |  |
| 1568807_a_at | 4.361371 | Hs.600720 | | | | | CDNA clone IMAGE:4825606 | | |  | |  |  |  |  |  |  |  |

**Table S2B**. List of genes induced by UV in SW13+Brg1 cells.

| **Probe Set ID** | **Fold change** | **Unigene(Avadis)** | **Gene Symbol** | **Gene Title** |
| --- | --- | --- | --- | --- |
| 202284_s_at | 2.758553 | Hs.370771 | CDKN1A | cyclin-dependent kinase inhibitor 1A (p21, Cip1) |
| 202672_s_at | 13.88431 | Hs.460 | ATF3 | activating transcription factor 3 |
| 202708_s_at | 2.038293 | Hs.2178 | HIST2H2BE | histone cluster 2, H2be |
| 202815_s_at | 2.470248 | Hs.15299 | HEXIM1 | hexamethylene bis-acetamide inducible 1 |
| 202859_x_at | 2.918234 | Hs.551925 | IL8 | interleukin 8 |
| 203725_at | 2.313093 | Hs.80409 | GADD45A | growth arrest and DNA-damage-inducible, alpha |
| 203821_at | 4.888228 | Hs.799 | HBEGF | heparin-binding EGF-like growth factor |
| 204621_s_at | 4.464731 | Hs.563344 | NR4A2 | nuclear receptor subfamily 4, group A, member 2 |
| 205193_at | 2.328492 | Hs.517617 | MAFF | v-maf musculoaponeurotic fibrosarcoma oncogene homolog F (avian) |
| 207064_s_at | 2.708629 | Hs.143102 | AOC2 | amine oxidase, copper containing 2 (retina-specific) |
| 207623_at | 2.559365 | Hs.654958 | ABCF2 | ATP-binding cassette, sub-family F (GCN20), member 2 |
| 211506_s_at | 2.266529 | Hs.551925 | IL8 | interleukin 8 |
| 212019_at | 2.054991 | Hs.401842 | RSL1D1 | ribosomal L1 domain containing 1 |
| 213988_s_at | 2.132653 | Hs.28491 | SAT1 | spermidine/spermine N1-acetyltransferase 1 |
| 214169_at | 2.145401 | Hs.438072 | UNC84A | unc-84 homolog A (C. elegans) |
| 220533_at | 2.157824 |  |  | Transcribed locus |
| 36711_at | 4.590843 | Hs.517617 | MAFF | v-maf musculoaponeurotic fibrosarcoma oncogene homolog F (avian) |
| 38037_at | 4.171355 | Hs.799 | HBEGF | heparin-binding EGF-like growth factor |
| 223218_s_at | 2.687203 | Hs.319171 | NFKBIZ | nuclear factor of kappa light polypeptide gene enhancer in B-cells inhibitor, zeta |
| 223666_at | 4.291938 | Hs.316890 | SNX5 | sorting nexin 5 |
| 228458_at | 2.088113 | Hs.571098 | LOC441150 | similar to RIKEN cDNA 2310039H08 |
| 228839_s_at | 2.087997 |  | LOC642361 | hypothetical gene supported by AF064843; AK025716 /// hypothetical LOC642361 |
| 228967_at | 2.705518 | Hs.150580 | EIF1 | Eukaryotic translation initiation factor 1 |
| 230304_at | 2.516872 | Hs.143408 | | CDNA clone IMAGE:30332316 |
| 230380_at | 3.411513 | Hs.245798 | THAP2 | THAP domain containing, apoptosis associated protein 2 |
| 230604_at | 2.111861 | Hs.598410 | | Transcribed locus |
| 231233_at | 2.344992 |  |  | Transcribed locus |
| 231417_at | 2.108866 | Hs.687784 | | Transcribed locus |
| 232097_at | 2.012649 | Hs.555910 | TOX4 | TOX high mobility group box family member 4 |
| 233376_at | 2.118903 | Hs.661713 | | G protein interaction factor 2-like mRNA sequence |
| 235174_s_at | 4.158825 | Hs.708094 | LOC100128822 | hypothetical protein LOC100128822 |
| 235587_at | 2.443015 | Hs.193172 | LOC202781 | hypothetical protein LOC202781 |
| 236124_at | 2.846526 | Hs.596513 | LOC153546 | hypothetical protein LOC153546 |
| 236213_at | 3.43566 | Hs.666143 | LOC100130885 | hypothetical protein LOC100130885 |
| 236875_at | 2.931401 | Hs.648517 | | Transcribed locus |
| 237116_at | 2.175351 | Hs.632559 | LOC646903 | hypothetical LOC646903 |
| 237510_at | 5.05185 |  | MYNN | myoneurin, Zinc-regulated transcription factor |
| 238012_at | 2.010369 | Hs.37916 | DPP7 | Dipeptidyl-peptidase 7 |
| 238228_at | 4.811037 |  |  | Transcribed locus, moderately similar to XP_001093747.1 PREDICTED: tumor protein |
| 238389_s_at | 3.035103 | Hs.222218 | | p53 binding protein, 2 [Macaca mulatta] |
| 238633_at | 2.777656 | Hs.167805 | EPC1 | Enhancer of polycomb homolog 1 (Drosophila) |
| 238646_at | 2.697884 | Hs.597327 | | Transcribed locus |
| 238813_at | 2.093773 | Hs.555936 | | Transcribed locus |
| 238946_at | 3.563391 | Hs.665649 | | Transcribed locus |
| 239266_at | 2.697571 | Hs.245367 | | Transcribed locus |
| 239536_at | 2.74662 | Hs.662791 | | Transcribed locus |
| 239814_at | 2.279929 | Hs.635013 | | Transcribed locus, strongly similar to XP_531062.1 PREDICTED: hypothetical protein |
| 242255_at | 3.569394 | Hs.709289 | LOC100130837 | Hypothetical protein LOC100130837 |
| 242594_at | 2.0656 | Hs.444517 | FAM44A | family with sequence similarity 44, member A |
| 243404_at | 3.461357 | Hs.443602 | | Transcribed locus |
| 243947_s_at | 2.089211 | Hs.120784 | | Transcribed locus |
| 1552362_a_at | 3.611434 | Hs.337588 | LEAP2 | liver expressed antimicrobial peptide 2 |
| 1554020_at | 2.240082 | Hs.505202 | BICD1 | bicaudal D homolog 1 (Drosophila) |
| 1554980_a_at | 3.915 | Hs.460 | ATF3 | activating transcription factor 3 |
| 1556213_a_at | 2.505789 | Hs.473420 | BTG3 | BTG family, member 3 |
| 1556216_s_at | 2.076457 | Hs.550108 | | CDNA clone IMAGE:5261375 |
| 1556346_at | 9.902552 | Hs.660628 | | Partial mRNA; ID YG39-1A |
| 1556588_at | 2.081757 | Hs.512015 | C15orf37 | chromosome 15 open reading frame 37 |
| 1557104_at | 3.249185 | Hs.464896 | ZNF397OS | Zinc finger protein 397 opposite strand |
| 1558256_at | 3.637448 | Hs.565253 | LOC148189 | hypothetical protein LOC148189 |
| 1560071_a_at | 4.183755 |  |  | Transcribed locus |
| 1560129_at | 2.067058 | Hs.684004 | | MRNA; cDNA DKFZp313H0240 (from clone DKFZp313H0240) |
| 1565759_at | 2.1287 | Hs.410817 | RPL13 | Ribosomal protein L13 |
| 1568807_a_at | 2.091842 | Hs.600720 | | CDNA clone IMAGE:4825606 |
